# Masquerade of Leukemic Infiltration With Cerebral Sinus Venous Thrombosis Causing Papilledema by Asparaginase Therapy

**DOI:** 10.7759/cureus.36274

**Published:** 2023-03-17

**Authors:** Abdulelah G Abumohssin, Ahmed N Alnabihi, Abdullah S Alqahtani

**Affiliations:** 1 Medicine and Surgery, Faculty of Medicine, King Abdulaziz University, Jeddah, SAU; 2 Medicine and Surgery, King Saud Bin Abdulaziz University for Health Sciences College of Medicine, Jeddah, SAU; 3 Ophthalmology, King Abdullah International Medical Research Center, Jeddah, SAU; 4 Ophthalmology, King Abdulaziz Medical City & National Guard Hospital, Jeddah, SAU; 5 Vitreoretinal and Ocular Oncology Surgery, King Saud Bin Abdulaziz University for Health Sciences, Jeddah, SAU

**Keywords:** chemotherapy, leukemic ophthalmopathies, retinal haemorrhage, leukemia, chemotherapy-related toxicity, asparaginase, cerebral sinus venous thrombosis, acute lymphoid leukemia

## Abstract

Acute lymphoblastic leukemia (ALL) is a hematological cancer that can cause ocular tissue involvement. Asparaginase is a chemotherapy regimen that is commonly used in leukemia which could lead to similar ocular manifestations. We report a patient with a history of ALL for seven months on asparaginase therapy and persistent cerebral sinus venous thrombosis (CSVT) with acute venous infarction in the left frontal lobe presented with worsening vision. On examination, he had a visual acuity (VA) of (6/21) in the right eye and (6/60) in the left eye, with a mild left eye abduction limitation. Fundal examination showed bilateral prominent multilayered retinal hemorrhages and papilledema with absence of leukemic infiltration. His chemotherapy regimen was held and a one month follow up was scheduled. Follow up after one month of chemotherapy cessation showed resolution of both VA and fundal exam findings. It is crucial to differentiate between asparaginase toxicity and infiltration of the disease in ALL patients. As this would determine whether the treatment should be continued or suspended.

## Introduction

Acute lymphoblastic leukemia (ALL) is a cancerous, hematologic, systemic disease of two types: B and T-cell [1]. ALL classically presents with general signs and symptoms of anemia, neutropenia, and thrombocytopenia. Furthermore, it could present with hepatosplenomegaly, lymphadenopathy, tender bones, and gum hypertrophy. Central nervous system involvement is common in ALL. Around 80% of cases of ALL presents in the pediatric age group; hence, ALL is considered the most common leukemia in this population [1-2].

Ocular tissue involvement could be observed in leukemic patients. Direct infiltration, hemorrhage, and ischemic changes are three common processes of ocular involvement [3]. The acute form appeared to affect the ocular tissue more frequently than chronic leukemia. In addition, standard chemotherapy regimens that are commonly used in leukemia could lead to ocular manifestations [4]. one of which is asparaginase, an anti-cancer medication used to treat ALL exclusively. Although it’s an uncommon side effect, asparaginase might result in cerebral sinus venous thrombosis (CSVT). Patients with CSVT usually present with intracranial hypertension, which could lead to certain signs and symptoms, such as papilledema, vomiting, and headache [5-6]. 

We report this case of a 15-year-old boy known case of ALL for seven months and left frontal lobe stroke for one week who presented with a complaint of bilateral gradual vision loss associated with double vision consistent with the presence of bilateral papilledema.

## Case presentation

A 15-year-old boy with a history of acute lymphoblastic leukemia for seven months and persistent cerebral sinus venous thrombosis with acute venous infarction in the left frontal lobe was referred to an ophthalmology department for evaluation of gradual worsening vision for one week. The patient's father reported no history of any eye surgeries, lasers, or injections. He has no history of eye traumas. There is a positive family history of diabetes mellitus, hypertension, pancreatic cancer, and hyperthyroidism. There is no family history of hematological diseases.

**Figure 1:**
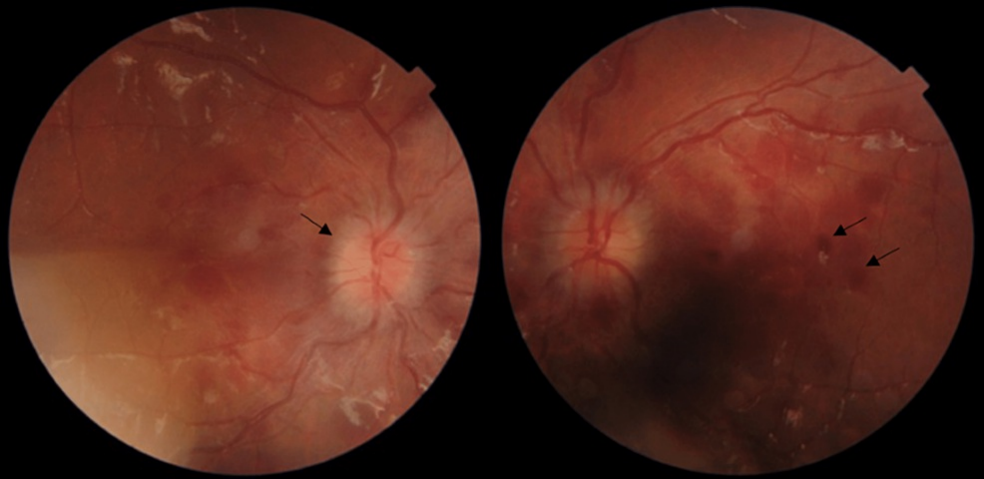
Colored fundus photo of both eyes The images are showing prominent multilayered retinal hemorrhages (arrows on the left eye) with papilledema (arrow on the right eye) before stopping asparaginase.

The patient had decreased visual acuity of (6/21) in the right eye and (6/60) in the left eye. His intraocular pressure was 15 mmHg in the right eye and 17 mmHg in the left eye. His pupil examination was normal. His extraocular muscle examination showed mild left eye abduction limitation. The fundus examination revealed the presence of bilateral disc swelling with scattered small multilayered retinal hemorrhages in both eyes (Figure 1). Other ocular assessments were within normal in both eyes.

**Figure 2:**
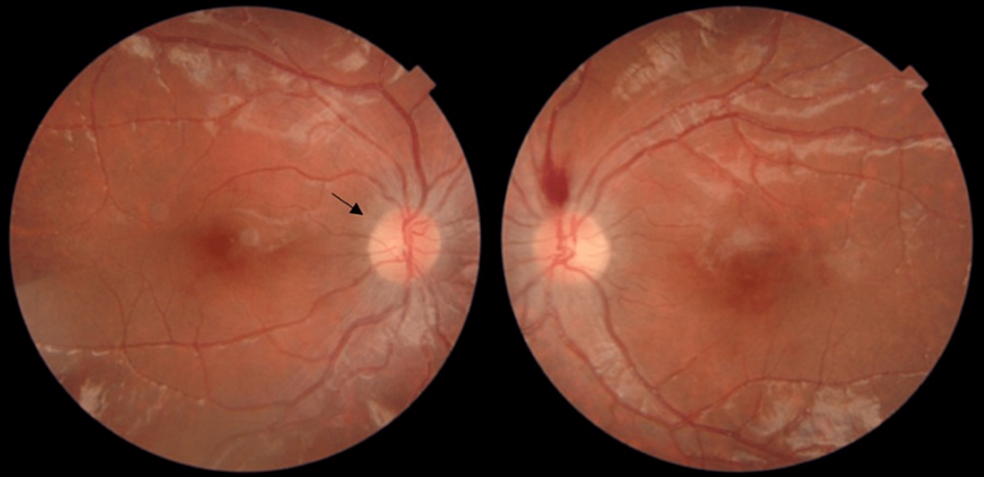
Colored fundus photo of both eyes one month after stopping the asparaginase Colored fundus photo of both eyes showing a resolution of the macular hemorrhages with the resolution of papilledema (arrow in the right eye) one month after stopping the asparaginase.

Upon admission the patient had a normal physical examination. Further investigations revealed asparaginase and steroid-induced hyperglycemia, Multifactorial hypertriglyceridemia, and pancreatitis. His complete blood count (CBC) was within normal limits. Fundal examination showed bilateral prominent multilayered retinal hemorrhages and papilledema with absence of leukemic infiltration. The patient’s asparaginase therapy was held and a one month appointment at the ophthalmology clinic was scheduled. During his follow up after one month of holding his antileukemic agent, the best corrected visual acuity (BCVA) in the right eye was (6/6), and in the left eye was (6/12). Furthermore, fundal exam showed bilateral resolution of multilayered retinal hemorrhages, as well as resolution of the papilledema (Figure 2). His left lateral abduction limitation was unresolved. Finally, the patient was shifted to a different modality of chemotherapy.

## Discussion

Retinal hemorrhage is a major complication that manifests in leukemic patients [7]. It might develop from direct leukemic infiltration, or secondary to factors that contribute to thrombophilia, for example, utilization of certain chemotherapeutic agents, elevated levels of coagulation factors VIII, IX and XI, elevated lipoprotein (a), and several other factors [8]. The risk of thrombosis increases by eight times if only one thrombophilia factor is present [9]. Our patient’s recent asparaginase treatment placed him at an increased risk of CVST, as asparaginase chemotherapy causes natural anticoagulant deficiency [10]. So, this shifts our minds from considering direct infiltration as the primary cause of retinal hemorrhage. Furthermore, our patient’s CBC was normal at the time of presentation which indicates a less likely diagnosis of leukemic infiltration. Instead, he experienced an obvious symptoms of asparaginase toxicity accompanied by worsening of vision, which led to ophthalmology clinic referral and treatment withdrawal. His fundal examination showed bilateral prominent multilayered retinal hemorrhages and papilledema with absence of leukemic infiltration. The patient was followed up after one month and his fundal exam showed bilateral resolution of multilayered retinal hemorrhages, as well as resolution of the papilledema.

Dubashi and Jain reported a similar case in which a 16-year-old male patient with ALL who developed cortical venous thrombosis manifesting as headaches, vomiting and multiple episodes of seizures three weeks after his exposure to asparaginase. The treatment was stopped as well, and a repeated CT brain was performed after one month of stopping the treatment showing normal results [5]. Similarly, Liu et al. stopped the asparaginase therapy when their patient developed severe thrombosis and signs of CVST [10]. Additionally, another study concluded that receiving less asparaginase therapy improves the outcomes substantially [11]. Depending on the degree of toxicity, asparaginase therapy maybe stopped or altered [12]. However, a study showed that even though asparaginase is associated with different toxicities, most of them are not an indication for discontinuation because they are nonfatal, reversible, and manageable [13]. Thus, the debate on whether or not to continue therapy on a lower dose after the recovery of the toxicity is yet to be further identified.

The importance of collaboration between ophthalmology-ocular oncology and oncology departments is crucial in differentiating between the leukemic infiltration and treatment side effects. In case of leukemic infiltration, treatment regimen should be continued though it is suspended in the incidence of treatment side effects. Retinal hemorrhage needs to be related to one of those etiologies and treated accordingly [14]. Finally, some well-known chemotherapy agents that cause vasculitis and thromboembolic cascade should be monitored during the period of therapy.

## Conclusions

In conclusion, this case highlights the importance in differentiating asparaginase toxicity from infiltration of the disease in ALL patients; thus history, sequence of medications, and good comprehension of the side effects is important. As this would determine whether the treatment should be continued or suspended. Cooperation between specialties and fast access is important to avoid diagnosis and management delay.
